# Blockage of Cholinergic Signaling via Muscarinic Acetylcholine Receptor 3 Inhibits Tumor Growth in Human Colorectal Adenocarcinoma

**DOI:** 10.3390/cancers13133220

**Published:** 2021-06-28

**Authors:** Nina A. Hering, Verena Liu, Rayoung Kim, Benjamin Weixler, Raoul A. Droeser, Marco Arndt, Ioannis Pozios, Katharina Beyer, Martin E. Kreis, Hendrik Seeliger

**Affiliations:** 1 Department of General and Visceral Surgery, Campus Benjamin Franklin, Charité Universitätsmedizin Berlin, 12200 Berlin, Germany; nina.hering@charite.de (N.A.H.); rayoung.kim@uksh.de (R.K.); benjamin.weixler@charite.de (B.W.); marco.arndt@charite.de (M.A.); ioannis.pozios@charite.de (I.P.); katharina.beyer2@charite.de (K.B.); martin.kreis@charite.de (M.E.K.); 2 Department of General Surgery, Vivantes Neukölln Hospital, 12351 Berlin, Germany; verena.liu@vivantes.de; 3 Department of Visceral Surgery, Clarunis University Center for Gastrointestinal and Liver Disease, St. Clara Hospital and University Hospital Basel, 4002 Basel, Switzerland; raoulandre.droeser@clarunis.ch

**Keywords:** colorectal cancer, acetylcholine, muscarinic acetylcholine receptor, cholinergic signaling, darifenacin, matrix metalloproteinase 1

## Abstract

**Simple Summary:**

Darifenacin is a selective muscarinic acetylcholine receptor 3 (M3R) antagonist in clinical use for benign bladder disorders. Here, the effects of M3R blockage by darifenacin on human colon adenocarcinoma cells are analyzed in vitro and in vivo. M3R expression was found in the majority of clinical colorectal cancer samples, making it attractive for drug targeting. In mice, darifenacin inhibited orthotopic and metastatic tumor xenograft growth. In vitro analyses on colon carcinoma cell lines showed that M3R targeting counteracts acetylcholine-induced p38, ERK1/2 and Akt signaling, and disrupts MMP-1 expression and tumor cell invasion. Our data suggest that targeting cholinergic signaling by darifenacin may be beneficial in the treatment of colorectal cancer.

**Abstract:**

Cholinergic signaling via the muscarinic M3 acetylcholine receptor (M3R) is involved in the development and progression of colorectal cancer (CRC). The present study aimed to analyze the blocking of M3R signaling in CRC using darifenacin, a selective M3R antagonist. Darifenacin effects were studied on HT-29 and SW480 CRC cells using MTT and BrdU assays, Western blotting and real time RT-PCR. In vivo, blocking of M3R was assessed in an orthotopic CRC xenograft BALB/c^nu/nu^ mouse model. M3R expression in clinical tumor specimens was studied by immunohistochemistry on a tissue microarray of 585 CRC patients. In vitro, darifenacin decreased tumor cell survival and proliferation in a dose-dependent manner. Acetylcholine-induced p38, ERK1/2 and Akt signaling, and MMP-1 mRNA expression were decreased by darifenacin, as well as matrigel invasion of tumor cells. In mice, darifenacin reduced primary tumor volume and weight (*p* < 0.05), as well as liver metastases, compared to controls. High expression scores of M3R were found on 89.2% of clinical CRC samples and correlated with infiltrative tumor border and non-mucinous histology (*p* < 0.05). In conclusion, darifenacin inhibited components of tumor growth and progression in vitro and reduced tumor growth in vivo. Its target, M3R, was expressed on the majority of CRC. Thus, repurposing darifenacin may be an attractive addition to systemic tumor therapy in CRC patients expressing M3R.

## 1. Introduction

Colorectal cancer (CRC) is a leading cause of cancer-related mortality [1]. While early and localized CRC is successfully treated surgically, median survival in metastatic disease is poor, in spite of new developments in systemic therapy. Recently, identification of molecular targets of the tumor and its microenvironment has contributed to a better understanding of the biology of CRC [2]. Interactions between neoplastic cells and autonomous and intrinsic gastrointestinal neurons play a major role in the microenvironment of CRC, as they orchestrate tumor progression [3].

Muscarinic acetylcholine (ACh) receptors are G protein-coupled receptors located in the membrane of the postganglionic parasympathetic nervous system. Among the receptor subtypes M1-M5 that are activated by ACh, the Gq-coupled M3 receptor (M3R) is widely expressed in gastrointestinal smooth muscle cells [4]. Its activation by ACh increases contraction of the bowel wall and thus gastrointestinal motility [4]. M3R is not only expressed on parasympathetic effector cells, which are part of the tumor microenvironment, but also on gastrointestinal neoplastic cells. More specifically, data have shown that M3R is expressed on human colon adenocarcinoma specimens and on CRC cell lines [5,6]. At the same time, CRC cells produce ACh themselves, thus forming an autocrine feed-forward loop [7]. In CRC, M3R acts as a conditional oncogene. Binding of ACh to M3R activates mitogenic signaling pathways which eventually leads to enhanced tumor cell growth, proliferation, survival, matrix metalloproteinase (MMP)-dependent membrane invasion and tumor cell migration and, finally, metastatic tumor spread [8,9,10]. In fact, ablation of M3R was shown to attenuate tumor development and growth in preclinical spontaneous or induced intestinal neoplasia models [11,12]. Earlier work from our group showed that vagotomy, and thus removal of the neuronal source of ACh, results in decreased tumor growth in APC^Min/+^ mice [13]. Thus, targeting M3R signaling may be an attractive therapeutic tool in controlling CRC.

Darifenacin (2, 3-dihydrobenzofuran-5-yl-ethyl-3-pyrrolidinyl-2, 2-diphenyl-acetamid) is an aromatic compound that is approved for clinical therapy of urinary incontinence in Europe and the United States, and it has a well-documented clinical safety profile [14]. As a competitive antagonist to muscarinic ACh receptors, it binds more than 20-fold more specifically to M3R than to other muscarinic receptors [15].

Our study aims to repurpose darifenacin towards M3R expressing CRC. We hypothesized that darifenacin is effective in attenuating biological effects of MR3 activation on early events of CRC progression on a cellular level in vitro, as well as tumor growth and metastasis in vivo. Further, we asked in which proportion M3R, as the biological target for darifenacin, is expressed on clinical CRC samples.

## 2. Results

### 2.1. M3R Is Expressed on CRC Cell Lines

**Figure 1:**
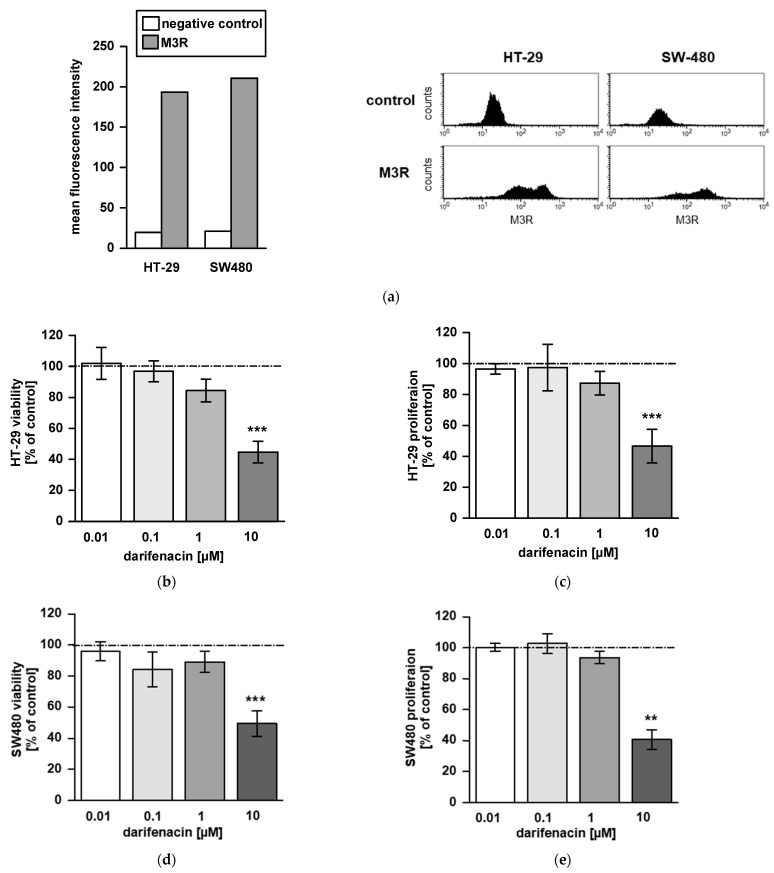
Darifenacin inhibits cell proliferation and survival of human colon carcinoma cell lines: (**a**) The presence of the muscarinic acetylcholine receptor 3 on HT-29 and SW480 cell lines was proved by fluorescent immunostaining and flow cytometry. For negative control, no primary antibody was added. The impact of darifenacin on cell viability and proliferation was tested by MTT and BrdU assays: (**b**) viability and (**c**) proliferation in HT-29 cells; (**d**) viability and (**e**) proliferation in SW480 and cells. In increasing concentrations, darifenacin reduced cell viability and proliferation in both cell lines (** *p* < 0.01; *** *p* < 0.001). Control values were set 100%, illustrated by the dotted line.

To study effects of the M3R antagonist darifenacin in vitro, we initially proved the presence of the M3 receptor on HT-29 and SW480 human colon carcinoma cell lines. In both cell lines, the receptor was stained and detected by flow cytometry (Figure 1a).

### 2.2. Darifenacin Inhibits CRC Cell Viability and Proliferation

We next analyzed the influence of darifenacin on cell viability and proliferation in MTT and BrdU assays. Darifenacin reduced viability and proliferation at a concentration of 10 µM in both cell lines HT29 (*p* < 0.001; Figure 1b,c) and SW480 (*p* < 0.01 and *p* < 0.001; Figure 1d,e). Lower concentrations were not effective. Though 1 µM of darifenacin slightly affected viability and proliferation in HT-29, this did not reach significance.

### 2.3. Darifenacin Inhibits ACh Induced p38, ERK1/2 and Akt Phosphorylation

**Figure 2:**
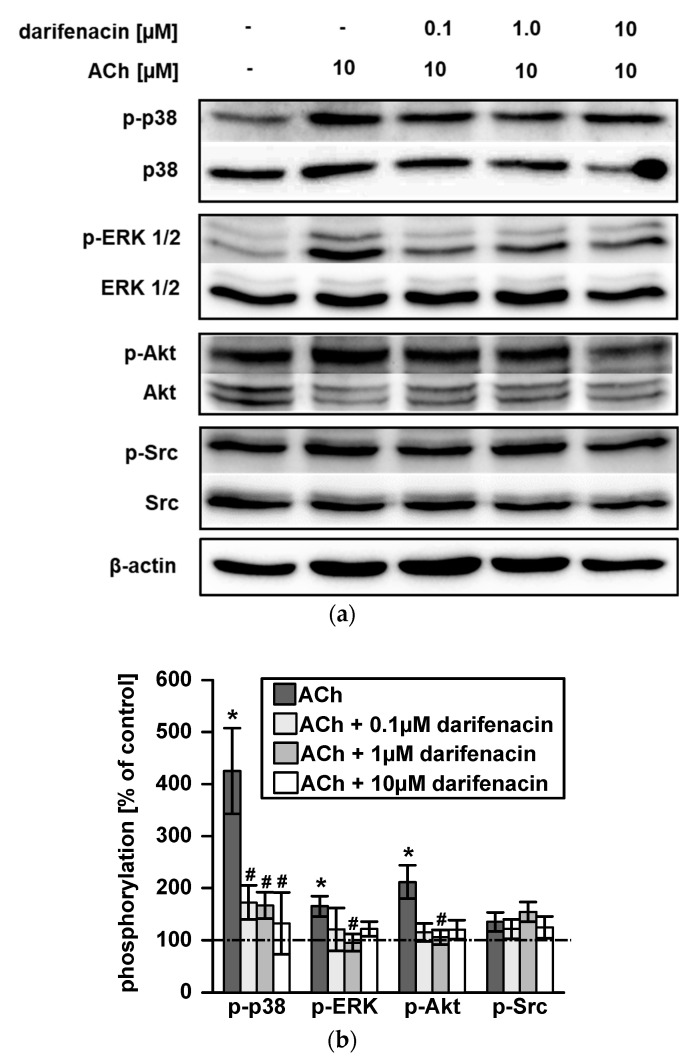
Inhibition of acetylcholine-activated signaling pathways by darifenacin in HT-29 cells: (**a**) Representative Western blots show acetylcholine-stimulated phosphorylation of p38, ERK 1/2 or Akt kinases and inhibitory effects of different concentrations of darifenacin (0.1, 1.0 and 10 µM). Full Western Blots are shown in Figure S1. (**b**) Densitometric quantification (* *p* < 0.05 vs. control; ^#^ *p* < 0.05 vs. ACh). Protein signal in control was set 100% (dotted line).

To test the influence of darifenacin on M3R post receptor signaling, Western blotting for phosphorylation of p38, ERK1/2, Akt and Src was performed. Representative Western blots of at least three independent experiments showed the phosphorylation levels of p38, ERK1/2, Akt and Src kinases with or without ACh stimulation and darifenacin treatment (Figure 2a). Challenging cells with 10 µM of ACh resulted in a three-fold increase in p38 phosphorylation and a two-fold induction of ERK1/2 and Akt phosphorylation compared to its untreated controls, respectively (*p* < 0.05; Figure 2b). Darifenacin inhibited ACh induced p38 phosphorylation at a low dose of 0.1 µM, which was as effective as 1 µM or 10 µM (*p* < 0.05; Figure 2b). ACh-stimulated phosphorylation of ERK1/2 and Akt was inhibited by 1 µM darifenacin (*p* < 0.05; Figure 2b). Although 0.1 and 10 µM darifenacin also seemed effective, quantification revealed no statistical significance (Figure 2b). Src phosphorylation was neither induced by 10 µM ACh nor affected by darifenacin (Figure 2a,b). Neither ACh nor darifenacin changed total levels of p38, ERK1/2, Akt or Src.

### 2.4. Darifenacin Inhibits ACh-Induced Invasion and MMP-1 Expression

**Figure 3:**
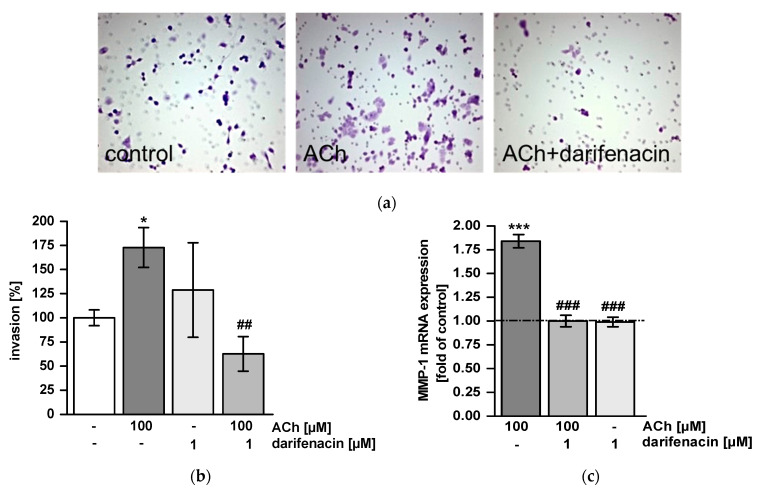
Darifenacin impedes acetylcholine-stimulated invasion of HT-29 cells: (**a**) Representative micrographs show HT-29 cells invaded into matrigel without stimulation (control), acetylcholine (ACh) stimulation or combined treatment with darifenacin and ACh (original magnification 200×). (**b**) Quantification of invaded cells. Darifenacin impeded ACh-stimulated invasion. Control values were set 100% (* *p* < 0.05 vs. control, ^##^ *p* < 0.01 vs. ACh). (**c**) Quantitative real time PCR showed enhanced MMP-1 gene expression in ACh-challenged cells, while darifenacin inhibited this upregulation (*** *p* < 0.001 ACh vs. control, ^###^ *p* < 0.001 darifenacin vs. ACh stimulation without darifenacin). mRNA levels are given as means ± SEM of increase over control. Control was set at 1-fold and is represented by the dotted line.

To test whether darifenacin inhibited tumor cell invasion, we used matrigel invasion chambers. ACh treatment stimulated invasion and enhanced the number of invaded cells compared to untreated controls (*p* < 0.05; Figure 3a,b). Pretreatment with darifenacin completely inhibited ACh-stimulated invasion (*p* < 0.01 vs. ACh; Figure 3a,b). Cells treated with both darifenacin and ACh were as invasive as untreated control cells (Figure 3b). In parallel, mRNA expression of MMP-1 was assessed by quantitative realtime PCR. ACh induced MMP-1 mRNA expression two-fold from the control level (*p* < 0.001 vs. control; Figure 3c). Darifenacin suppressed this ACh-stimulated induction completely at concentrations of 1 µM and 10 µM (*p* < 0.001 vs. ACh, Figure 3c).

### 2.5. Darifenacin Inhibits Orthotopic and Metastatic Tumor Growth In Vivo

**Figure 4:**
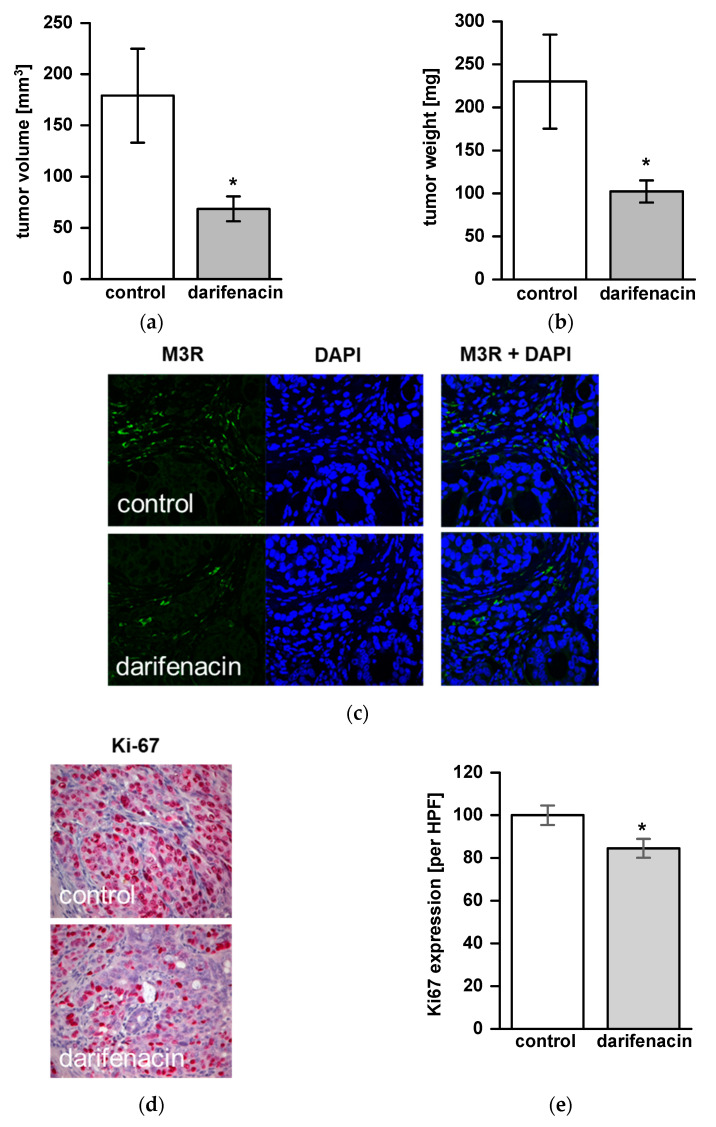
Darifenacin inhibits tumor growth in a xenograft model of human colon adenocarcinoma. Mice were treated with darifenacin or saline for 42 days after orthotopic tumor cell injection. After removing tumors, (**a**) tumor volume and (**b**) weight were determined for each animal and mean values ± SEM were calculated for each group (* *p* < 0.05 vs. control). (**c**) The presence of human muscarinic acetylcholine receptor 3 (M3R) in primary tumors of control and darifenacin-treated animals was confirmed by immunofluorescence staining. Negative control was performed without primary antibody. Representative slides show M3R in green and nuclei in blue (original magnification 400×). (**d**) Tumor cell proliferation was significantly reduced in darifenacin-treated animals compared to controls (* *p* < 0.05). (**e**) Ki-67 immunohistochemistry of darifenacin treated mice and controls (original magnification 400×).

Due to its inhibitory effects on cell viability, proliferation, ACh signaling and invasion in vitro, the impact of darifenacin was studied in a xenograft mouse model. Twelve animals were examined in both the treatment group and the control group. One animal died during the observation period. Tumor growth was detected in 21 of 23 animals (91%) at the end of the experiment. Overall, 10 animals in the treatment group and 11 animals in the control group were analyzed. Mean tumor size was 68.5 mm^3^ ± 12.8 in the treatment group vs. 179.1 mm^3^ ± 48.2 in the control group (*p* < 0.05; Figure 4a). Mean tumor weight was 102.2 ± 13.4 mg in the treatment group vs. 230.1 ± 57.5 mg in the control group (*p* < 0.05, Figure 4b). In the treatment group, lymph node metastases were found in 6 out of 10 animals (60%) and in 10 out of 11 animals (90%) of the control group. Liver metastases were not found in the treatment group but were detected in one animal (9%) of the control group. Neither in the control group nor in the darifenacin group, we detected a significant loss of body weight during the treatment period. M3R was expressed in vivo in both groups without a quantitative difference between darifenacin-treated animals and controls (Figure 4c). In vivo, proliferation was reduced significantly in darifenacin-treated animals (84.5% Ki-67 immunoreactive tumor cells per HPF, compared to controls, *p* < 0.05, Figure 4d,e).

### 2.6. M3R Is Expressed on Human CRC Samples and Correlates with Infiltrative Tumor Border Configuration and a Non-Mucinous Histological Subtype

**Figure 5:**
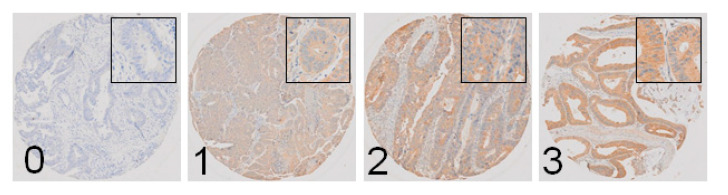
Muscarinic acetylcholine receptor 3 (M3R) is expressed in human colorectal cancer specimens. Representative slides of specimens with no (0, *n* = 9), low (1, *n* = 54), intermediate (2, *n* = 317) and high (3, *n* = 205) cytoplasmatic immunostaining. M3R expression scores of 2 and 3 were seen in 89.2% of patients (original magnification 40-fold, inserts original magnification 400×).

Epidemiological and clinicopathological data of 585 CRC patients analyzed for M3R expression are presented in Table 1. High M3R expression was found in 522 patients (89.2%). When M3R expression was correlated with clinicopathological features in univariate analyses, infiltrative tumor border configuration and a non-mucinous histological subtype showed a trend towards correlation with M3R expression that was not statistically significant (*p* = 0.080, and *p* = 0.070, respectively, Table 2). In multivariate regression analysis, infiltrative tumor border configuration and a non-mucinous histologic subtype were significantly correlated with M3R expression (*p* = 0.024 and *p* = 0.011, respectively, Table 2). In both analyses, age, sex, tumor location, pT status, pN status, grading, vascular invasion and microsatellite instability did not correlate with M3R expression. Representative histological specimens are shown in Figure 5.

## 3. Discussion

Within the present study we investigated the influence of darifenacin, a selective antagonist of the M3R subtype of muscarinic ACh receptors, on M3R-dependent mechanisms involved in the development and progression of CRC. As the most important finding, the results presented here demonstrate that darifenacin inhibits tumor cell proliferation and viability in vitro and reduces tumor growth and metastatic spread in an orthotopic xenograft mouse model. Cell culture experiments identified darifenacin as a potent antagonist of ACh-induced signaling pathways through inhibition of p38, ERK1/2 and Akt phosphorylation, and it impedes ACh-stimulated MMP-1 expression and cell invasion in vitro. In addition, the expression of M3R was clearly demonstrated in tumor samples of CRC patients.

Neural input into the tumor microenvironment has recently emerged as a major contributor to tumor progression that is caused by bidirectional signaling crosstalk of epithelial tumor cells and neural cells and their glia [3,16,17,18]. In a clinical context, perineural invasion (PNI) has been identified as a risk factor for poor prognosis in CRC and other gastrointestinal cancers [19,20]. On the other hand, parasympathetic input by ACh via its muscarinic receptor M3R contributes relevantly to CRC carcinogenesis and progression [13,21]. Indeed our in vitro and TMA experiments, in line with recent data, show that cytoplasmic and membrane M3R expression are present in CRC. Colon adenomas express higher levels of M3R compared to normal epithelium, suggesting M3R expression is an early feature in colorectal neoplasia [5]. Apart from ACh release by neurons and enteric neuroepithelial cells, ACh is expressed and secreted by CRC cells, resulting in an autocrine activation loop [7,22]. ACh itself is synthetized from the transfer of an acetyl group from acetyl-coenzyme A to choline by choline acetyltransferase (ChAT). ChAT itself is overexpressed in colon cancer cells compared to normal mucosa [7], while ChAT immunoreactive tuft cells are found throughout the gastrointestinal tract [10,23].

Our data show that darifenacin has antiproliferative and antisurvival effects on CRC in vitro and in vivo. This is in line with other studies that described darifenacin reducing tumor growth in xenograft models of gastric [24] and small cell lung cancer [25]. Since ACh stimulates ERK and Akt signaling pathways, two major contributors of tumor cell proliferation, it is no surprise that counteracting ACh with darifenacin inhibits proliferation [26]. Despite a significant effect, darifenacin did not completely abolish tumor cell proliferation in vivo in our experiments. Again, this was not unexpected, since multiple M3R independent cell proliferation pathways are active in vivo. In this regard, recent clinical data suggest that M3R signaling preferentially influences early events in tumor development, such as proliferation, invasion and migration [5].

We were able to show that ACh drives tumor cell invasion and migration, at least in part via an overexpression of MMP-1. This is in line with the data of Raufman’s group, who showed that M3R agonists stimulate MMP-1-dependent invasion and migration [27]. Darifenacin antagonizes these effects, in a similar manner to other selective or non-selective muscarinic antagonists or ablation of the gene coding for M3R *(CHRM3)*, or *EGFR* and *MMP1* genes [28]. Since the ability of tumor cells to migrate, penetrate basal membranes and invade lymph or blood vessels is a requirement for metastatic tumor spread [29], it is plausible that darifenacin may be able to reduce metastases. This is in concordance with clinical data of MMP-1 overexpression correlating strongly with advanced tumor stage, metastatic progression and adverse outcomes in colon cancer [5,30,31].

Cholinergic agonists bind to M3R, activate PKC/p38 and transactivate the EGF receptor. In our experiments, p38 phosphorylation following ACh treatment was by far more intense than Akt and ERK1/2 phosphorylation. This may be explained by the fact that HT-29 harbors a BRAF^V600E^ mutation, which constitutively activates Akt-, ERK- and Src-dependent signaling, so transactivation of the EGF receptor by ACh does not relevantly add to phosphorylation of the proteins downstream of the EGF receptor. In contrast, EGF receptor signaling independent of ACh via PKC/p38 is not influenced by the BRAF mutation, so ACh has a greater effect on this signaling pathway. In contrast, NIH-H508 cells have an inactivating heterozygous BRAF^G596R^ mutation and intact EGFR signaling [32]. In this cell line, ACh stimulated ERK1/2 phosphorylation, which was abolished by atropine [33]. Accordingly, darifenacin antagonized ACh-dependent activation of ERK1/2, Akt and p38 kinases in our experiments. The p38 MAP kinase was identified as a key player in M3R-induced MMP-1 gene induction [34]. Simultaneous activation of PKC/p38 and EGFR/ERK signaling potentiates MMP-1 gene and protein expression, and augments colon cancer cell invasion while blocking MMP-1 activity abolishes M3R agonist-induced colon cancer cell invasion [34].

M3R-dependent signaling has been shown to promote tumor growth and progression, not only in CRC but in non-small cell lung cancer and gastric cancer [24,35,36]. Interestingly, recent data from Renz et al. demonstrate that disruption of ACh signaling by vagotomy or muscarinic ACh receptor 1 (*Chrm1*) ablation accelerated tumorigenesis in experimental pancreatic cancer [37]. Accumulating these data on cholinergic signaling suggests differential organ- and receptor-specific effects on tumorigenesis that need further explanation.

To validate our in vitro results, we used an orthotopic xenograft mouse model. We injected human CRC cells into nude mice and found a potent reduction in primary tumor growth by darifenacin. Others have previously reported that treatment of APC^Min/+^ mice with the muscarinic receptor antagonist scopolamine butylbromide reduced the number of small bowel tumors [11]. Additionally, genetic ablation of M3R signaling resulted in attenuated tumor formation in M3R-deficient mice treated with azoxymethane, or double transgenic APC^Min/+^M3R^−/−^ mice, to induce colonic tumors [12]. Our model differs from the reported data in that we (1) treated an orthotopic human tumor xenograft as opposed to preventing tumorigenesis, and (2) used a drug instead of *CHRM3* ablation, simulating a scenario that is more similar to the situation of CRC patients. Nevertheless, our results are in line with the published in vivo data and add to the body of evidence that antagonizing M3R not only prevents de novo tumor formation but may indeed be a tool to treat CRC in a clinical setting. Finally, since bile acids present intraluminally promote CRC tumorigenesis, antagonizing bile acid-induced M3R activation may add to the benefit of darifenacin [38,39].

In the clinical cohort examined, M3R was strongly expressed in almost 90 percent of patients, which is consistent with the findings of others in clinical CRC specimens and cell lines [5,6]. Tumors showing an infiltrative border configuration had a higher expression of M3R, compared to a pushing tumor border configuration. This observation seems plausible and also is in line with our in vitro experiments that show a higher MMP-1 expression and infiltrative capabilities of tumor cells after ACh treatment. Further, MMP-1 expression has been reported to correlate with an infiltrative growth pattern in human CRC specimens [40]. As a second finding, though mucinous adenocarcinoma seems underrepresented in our cohort, non-mucinous CRC showed a slight but significantly higher presence of M3R expression than mucinous CRC. Present data on mucinous CRC is sketchy, and there is no consensus whether the presence of a mucinous histological type correlates with a higher biological aggressiveness in CRC [41]. Recent data suggest that mucin expression correlates with the presence of PNI [42], but whether there is a molecular link between mucin expression and M3R expression remains to be elucidated. In contrast, loss of MUC-2 expression and PNI were independent predictors for a poor survival in CRC patients [43]. However, clinical evidence is lacking as to whether cholinergic signaling is present in CRC patients with PNI, as it is in pancreatic ductal adenocarcinoma [44]. In CRC, PNI correlates with poor survival, and rectal cancer patients with PNI benefit from adjuvant chemotherapy [45,46]. While it seems plausible, evidence is lacking in terms of a possible correlation between PNI, M3R expression and cholinergic signaling in CRC. This would be a rationale for adding darifenacin to standard postoperative chemotherapy in CRC patients with PNI. Furthermore, recent epidemiological data show that patients treated with antimuscarinic medications for urological indications had a significantly reduced incidence of CRC [47]. Finally, oxaliplatin, which is used routinely in the treatment of CRC, induces a significant reduction in sensory and adrenergic innervations, as well as the total number and proportion of ChAT immunoreactive neurons [48]. Whether this effect adds to the cytotoxicity of oxaliplatin is unclear.

Our study has some limitations that should be mentioned. While an orthotopic xenograft mouse model is able to simulate a tumor microenvironment more realistically than non-orthotopic in vivo models, we used a nude mouse model in which a T cell deficiency exists. As T cells are capable of synthesizing ACh and express nicotinic and muscarinic ACh receptors, it would be interesting to know whether T cells contribute to muscarinic signaling in a tumor context [49]. Further, immunologic and inflammatory host responses may be different from syngeneic models. On the other hand, the model enabled us to observe human (as opposed to mouse) CRC cell growth in vivo.

In our TMA data, we found that M3R is expressed in the majority of CRC patients. While M3R is the target for darifenacin, we have no information as to whether M3R is activated by ACh or other ligands in the clinical specimens. Further, since PNI was not assessed systematically in our cohort, we were not able establish a correlation between M3R expression and PNI.

## 4. Materials and Methods

### 4.1. Cell Lines, Cell Culture and Reagents

Human colon cancer cells lines HT-29 and SW480 were obtained from the American Type Culture Collection (Rockville, MD, USA). Cells were cultivated as subconfluent monolayers in an incubator under standard conditions (37 °C, steam-saturated atmosphere, 5% CO_2_) in RPMI-1640 medium (Life Technologies, Darmstadt, Germany), containing 10% fetal calf serum and 1% penicillin and streptomycin (all Biochrom, Berlin, Germany). Darifenacin and ACh were purchased from Sigma-Aldrich (Schnelldorf, Germany).

### 4.2. Measurement of Cell Viability and Proliferation

Cells were seeded into 96-well microplates in sextuplicate (3 × 10^3^ cells per well). After incubation at 37 °C for 24 h, cells were shifted to serum-free conditions and were treated with darifenacin (0.01, 0.1, 1, and 10 µM) for 48 h. Then, cell viability and proliferation were assessed by MTT (3-[4,5-dimethylthiazol-2-yl]-2, 5-diphenyltetrazolium bromide; Sigma-Aldrich) and BrdU (5-bromo-2′-deoxyuridine; Roche, Mannheim, Germany) assays, respectively, according to the manufacturers’ protocols.

### 4.3. Flow Cytometry

Cells were detached, fixed with 4% paraformaldehyde for 10 min at 4 °C, permeabilized with 0.1% Tween and blocked with donkey serum (Jackson ImmunoResearch, Cambridge, UK). Staining was carried out with an anti-M3R antibody (1:200; ab154835, Abcam, Cambridge, UK) and rabbit-Alexa Fluor 488 labeled secondary antibody (1:250; A21206, Thermo Fisher Scientific, Darmstadt, Germany). As a negative control, staining was performed without the primary antibody. Stained cells were analyzed and counted by subsequent flow cytometry (FACSCalibur, BD Biosciences, Heidelberg, Germany).

### 4.4. Western Blotting

Cells were pretreated with darifenacin (0.1, 1 and 10 µM) for 60 min, followed by addition of ACh (10 µM) for 5 min. Following treatment with ACh, darifenacin or both, cells were lysed using radioimmunoprecipitation assay lysis buffer (Thermo Fisher Scientific) containing phosphatase and protease inhibitors. Protein concentration was quantified by a Quantipro BCA protein assay kit (Sigma-Aldrich) according to the manufacturer’s protocol. Cell extracts were separated by polyacrylamide gel electrophoresis and blotted on polyvinylidene fluoride membranes (Perkin Elmer, Boston, MA, USA). For immune detection, the following primary antibodies were used: anti-p38 (8690), anti-phospho-p38 (4511), anti-ERK1/2 (4695), anti-phospho-ERK1/2 (4370), anti-Akt (pan, 4685), anti-phospho-Akt (pan, 9018), anti-Src (2109S), anti-phospho-Src (6943S; all antibodies 1:1000, Cell Signaling, Leiden, Netherlands) and anti-β-actin (A5441, Sigma-Aldrich). Bound antibodies were visualized by luminescence imaging (Fusion FX, Vilber, Marne-la-Vallée, France) using peroxidase-conjugated secondary antibodies (A9169 and A9044, Sigma-Aldrich) and a chemiluminescent substrate system (SuperSignal West Pico PLUS, Thermo Fisher Scientific). Densitometric quantification was carried out with Image J. β-actin served as internal loading control.

### 4.5. Cell Invasion Assay

Cell invasion was analyzed using Biocoat Matrigel Invasion Chambers (Corning, Wiesbaden, Germany). After cultivating HT-29 cells in serum-free medium overnight, 3 × 10^5^ cells were seeded into the upper chamber of each transwell insert. Darifenacin (1 µM) was added one hour before treatment with ACh (100 µM). Both substances were placed to the upper and lower chamber; FBS was added to the lower chamber only and served as chemoattractant. After an incubation period of 48 h at 37 °C, non-invading cells were removed. Invaded cells were fixed, stained with 0.2% crystal violet (Sigma-Aldrich) and visualized by light microscopy. Samples were blinded and cells were counted in 16 consecutive areas of each specimen (20× magnification).

### 4.6. Quantitative Real Time RT-PCR

Cells were treated with darifenacin (1 µM) for one hour, followed by an incubation with ACh (100 µM) for additional four hours. RNA was extracted using NucleoSpin^®^ RNA (Macherey-Nagel, Düren, Germany) and RT-PCR was carried out with a High-Capacity cDNA Archive Kit (Thermo Fisher Scientific) according to the manufacturer’s protocol. For quantitative real time PCR, a TaqMan Gene Expression Assay for MMP-1 (Hs00899658_m1) was used, while S9 ribosomal protein (Hs00396989_m1) served as endogenous control (both Thermo Fisher Scientific). PCR was set up in duplicate, and gene expression was calculated from threshold cycle values according to the 2^−ΔΔCt^ method [50].

### 4.7. Orthotopic Tumor Xenograft Mouse Model

Male BALB/c^nu/nu^ nude mice (6–10 weeks old, body weight 20–25 g) were purchased from Janvier Labs (Le Genest-Saint-Isle, France). Animals were kept under a 12 h/12 h day/night cycle with free access to food and water. All animal experiments were approved by the regional authority (Landesamt für Gesundheit und Soziales, Berlin, Germany, G0136/14) and performed in compliance with the European Union guideline 2010/63/EU.

Orthotopic cellular tumor xenografting was carried out as described before [51]. Briefly, following anesthesia with ketamine, xylazin (both Pfizer, Berlin, Germany) and laparotomy, 10 µL of cell suspension containing 10^6^ HT-29 cells was injected subserosally into the cecal wall. The abdomen was closed with interrupted sutures.

Starting two days after tumor cell injection, animals received a daily intraperitoneal injection of darifenacin (2.5 mg per kg body weight in 0.1 mL NaCl; Sigma-Aldrich) or vehicle (0.1 mL NaCl). All animals were monitored daily for general condition, weight loss, and for abundant tumor growth. They were anesthetized and sacrificed after 42 days, and primary tumor, lymph nodes and liver metastases were removed. All primary tumors were measured using a caliper and weighed. The tumors were fixated and embedded for immunofluorescence staining.

### 4.8. Immunofluorescence

Embedded tumor sections were cut (4 µm), deparaffinized and rehydrated. For epitope retrieval, sections were heated in sodium citrate solution at pH 6.0. Afterwards, slides were permeabilized with 0.5% Triton X-100 and blocked with 1% bovine serum albumin and 5% goat serum. Immunostaining of M3R was carried out with 1:100 anti-CHRM3 (HPA024106, Sigma-Aldrich) overnight at 4 °C, followed by incubation with labelled Alexa Fluor 488 goat anti-rabbit IgG (A21206, Thermo Fisher Scientific) for two hours at room temperature. Nuclei were stained with 4′, 6-Diamidin-2-phenylindol (DAPI, 1:5000, Sigma-Aldrich). Imaging was performed by confocal laser scanning microscopy (LSM 780, Zeiss, Jena, Germany).

### 4.9. Immunohistochemistry

Tissue sections were prepared and treated as described for immunofluorescence staining. Staining was performed with anti-Ki67 primary antibody (9027, Cell Signaling) at 4 °C overnight followed by biotin-conjugated polyclonal swine anti-rabbit secondary antibody (E0353, Agilent Technologies, Santa Clara, CA, USA) and phosphatase-conjugated streptavidin (Seracare Life Sciences, Milford, CT, USA). Signals were visualized by HistoMark^®^ RED (Seracare Life Sciences) and hematoxylin counterstaining (Sigma-Aldrich). For quantification, Ki67-positive cells were counted per high power field (HPF = 0.159 mm^2^, 10 HPF counted). The mean of Ki67-positive cells was calculated and expressed in percent.

### 4.10. Tissue Microarray Construction and Analysis

A tissue microarray (TMA) was constructed from biopsy specimens of 585 primary CRC patients. Clinical data of the patients were retrieved from a prospectively maintained database. Briefly, formalin-fixed, paraffin-embedded tissue blocks were prepared according to standard procedures. Tissue cylinders with a diameter of 0.6 mm were punched from morphologically representative areas of each formalin-fixed, paraffin-embedded donor block and brought into one recipient paraffin block using a semiautomated tissue arrayer. Each punch was made from the center of the tumor, so that each TMA spot consisted of at least 50% tumor cells. Immunohistochemical staining was performed on a Benchmark immunohistochemistry staining system (Leica Biosystems, Muttenz, Switzerland) with bond polymer refine detection solution (Leica Biosystems) for 3, 3′-diaminobenzidine using an anti-MR3 primary antibody (1:100, AHP1355, Biorad Laboratories, Neuberg, Germany). Antigen retrieval was performed using citrate solution at pH6 for 30 min at 95 °C. M3R staining intensity was scored from 0 (no reaction) to 3 (strong reaction). Low expression of M3R was defined as scores of 0 and 1, and high expression was defined as scores of 2 and 3. Scoring was performed by two independent pathologists blinded for the clinical data. Experiments using human material and data were approved by the regional ethics committee (Ethikkommission beider Basel, EK120/13).

### 4.11. Statistical Analysis

Descriptive statistics include mean ± standard error of the means. Statistical significance was assessed with the two-side unpaired Student’s *t*-test and adjusted according to Bonferroni–Holm in case of multiple testing. For clinical data, categorical variables were reported as frequencies (numbers and percentages) and continuous variables as means ± standard deviations. The primary endpoint of M3R expression was analyzed by univariate and multivariate logistic regression analysis. In the multivariate logistic regression, possible confounders were adjusted for. Chi-square tests were used to analyze correlations between categorical variables. *p* values less than 0.05 were considered statistically significant. All statistical analyses were performed with STATA 13 (StataCorp, College Station, TX, USA).

## 5. Conclusions

M3R is expressed in the majority of clinical CRC specimens, and the M3R antagonist darifenacin inhibits components of CRC progression in vitro and in vivo. The involvement of M3R in the regulation of cell survival, proliferation and migration suggests that M3R antagonists may be effective in CRC treatment or chemoprevention. Since darifenacin has a proven safety profile in humans, it may be an attractive addition to conventional chemo-therapy in CRC patients.

## Figures and Tables

**Table 1:** Muscarinic acetylcholine receptor 3 (M3R) expression in 585 primary colorectal cancer patients having undergone primary surgery. M3R expression scores of 2 and 3 were considered as high. Percentages are given as percent of the entire cohort and the subgroups, respectively. *^a^* Age is given as mean and range; *^b^* missing data.

Parameter	*n*	M3R Low	M3R High
Total	585	63 (10.8%)	522 (89.2%)
Age [years]	68.6 (30–96) *^a^*	67.2 (30–87) *^a^*	68.8 (36–96) *^a^*
Sex			
Female	322 (55.0%)	32 (9.9%)	290 (90.1%)
Male	263 (45.0%)	31 (11.8%)	232 (88.2%)
Tumor location *^b^*			
Left sided	405 (69.2%)	41 (10.1%)	364 (89.9%)
Right sided	178 (30.4%)	22 (12.4%)	156 (87.6%)
Histologic subtype			
Mucinous	28 (4.8%)	6 (21.4%)	22 (78.6%)
Non-mucinous	557 (95.2%)	57 (10.2%)	500 (89.8%)
pT status *^b^*			
pT1/2	111 (19.0%)	14 (12.6%)	97 (87.4%)
pT3/4	464 (79.3%)	44 (9.5%)	420 (90.5%)
pN status			
pN0	311 (53.2%)	32 (10.3%)	279 (89.7%)
pN1-2	274 (46.8%)	31 (11.3%)	243 (88.7%)
Grading *^b^*			
G1/2	559 (95.6%)	55 (9.8%)	504 (90.2%)
G3	16 (2.7%)	3 (18.8%)	13 (81.3%)
Vascular invasion *^b^*			
Present	161 (27.5%)	19 (11.8%)	142 (88.2%)
Absent	414 (70.8%)	39 (9.4%)	375 (90.6%)
Tumor border configuration *^b^*			
Infiltrative	396 (67.7%)	46 (11.6%)	350 (88.4%)
Pushing	58 (9.9%)	12 (20.7%)	46 (79.3%)
Microsatellite instability			
Deficient	68 (11.6%)	8 (11.8%)	60 (88.2%)
Proficient	517 (88.4%)	55 (10.6%)	462 (89.4%)

**Table 2:** Correlation of muscarinic acetylcholine receptor 3 (M3R) expression with clinicopathological parameters in 585 primary colorectal cancer patients. In univariate analyses, no parameter correlated with M3R expression. In multivariate analysis, M3R expression correlated significantly with infiltrative tumor border configuration and non-mucinous histologic subtype. OR, odds ratio; CI, confidence interval.

Parameter	Univariate Regression		Multivariate Regression	
	OR (95% CI)	*p* Value	OR (95% CI)	*p* Value
Age				
<60	Reference		Reference	
>=60	1.29 (0.72–2.32)	0.381	1.01 (0.98–1.04)	0.438
Sex				
Female	Reference		Reference	
Male	0.83 (0.49–1.39)	0.473	0.75 (0.42–1.32)	0.315
Tumor location				
Right sided	Reference		Reference	
Left sided	0.80 (0.46–1.39)	0.424	0.77 (0.41–1.44)	0.415
Histologic subtype				
Non-mucinous	Reference		Reference	
Mucinous	0.418 (0.16–1.07)	0.070	0.26 (0.10–0.74)	0.011
pT status				
pT1/2	Reference		Reference	
pT3/4	1.38 (0.73–2.61)	0.327	1.89 (0.88–4.03)	0.102
pN status				
pN0	Reference		Reference	
pN1–2	0.90 (0.53–1.52)	0.690	1.03 (0.55–1.92)	0.926
Grading				
G1	Reference		Reference	
G2	2.43 (0.67–8.87)	0.177	1.98 (0.48–8.12)	0.346
G3	0.67 (0.16–2.84)	0.558	0.49 (0.10–2.55)	0.399
Vascular invasion				
Present	Reference		Reference	
Absent	0.77 (0.43–1.39)	0.396	1.02 (0.52–2.02)	0.956
Tumor border configuration				
Infiltrative	Reference		Reference	
Pushing	0.55 (0.29–1.07)	0.080	0.43 (0.20–0.89)	0.024
Microsatellite instability				
Deficient	Reference		Reference	
Proficient	1.12 (0.51–2.47)	0.778	0.96 (0.40–2.28)	0.928

## Data Availability

All data supporting the findings of this study are available within this paper and from the corresponding author.

## Supplementary Materials

The following are available online at https://www.mdpi.com/article/10.3390/cancers13133220/s1, Figure S1: Blots relative to Figure 2a.
