# Supplementary material for: Blockage of Cholinergic Signaling via Muscarinic Acetylcholine Receptor 3 Inhibits Tumor Growth in Human Colorectal Adenocarcinoma
Source: Cancers (Basel). 2021 Jun 28;13(13):3220. doi: 10.3390/cancers13133220 (PMC8267754; doi:10.3390/cancers13133220)
Supplement: Supplementary file 1 [file cancers-13-03220-s001.zip › cancers-1250956-supplementary.pdf]

cancers MDPI

Supplementary Material: Blockage of Cholinergic Signaling via Muscarinic Acetylcholine Receptor 3 Inhibits Tumor Growth in Human Colorectal Adenocarcinoma

Nina A. Hering, Verena Liu, Rayoung Kim, Benjamin Weixler, Raoul A. Droeser, Marco Arndt, Ioannis Pozios, Katharina Beyer, Martin E. Kreis and Hendrik Seeliger

2018-01-19

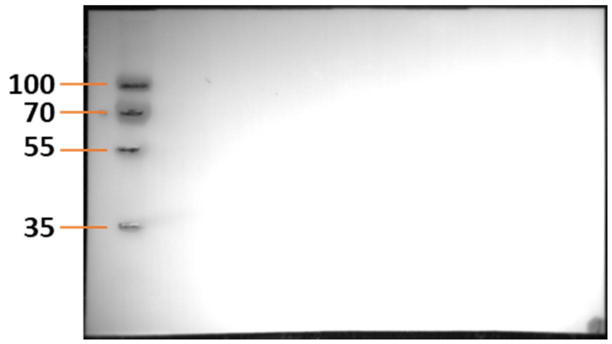

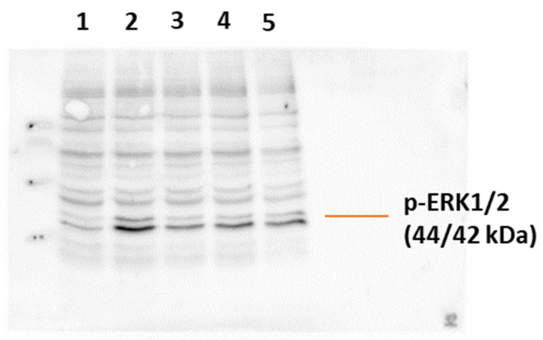

ERK

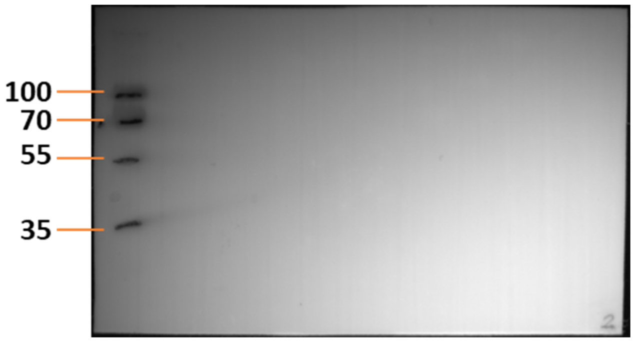

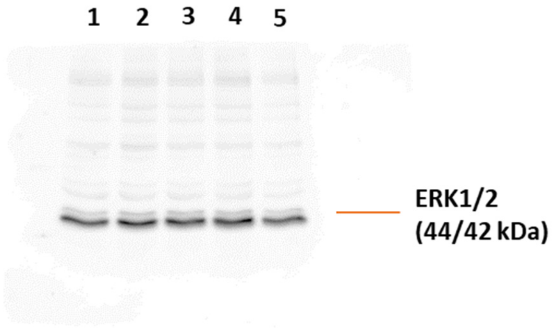

β-actin

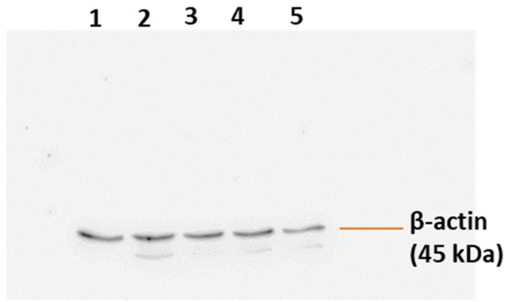

1 untreated control
2 10 µM Ach
3 0.1 µM darifenacin + 10 µM Ach
4 1 µM darifenacin + 10 µM Ach
5 10 µM darifenacin + 10 µM Ach

2018-02-19

p-ERK

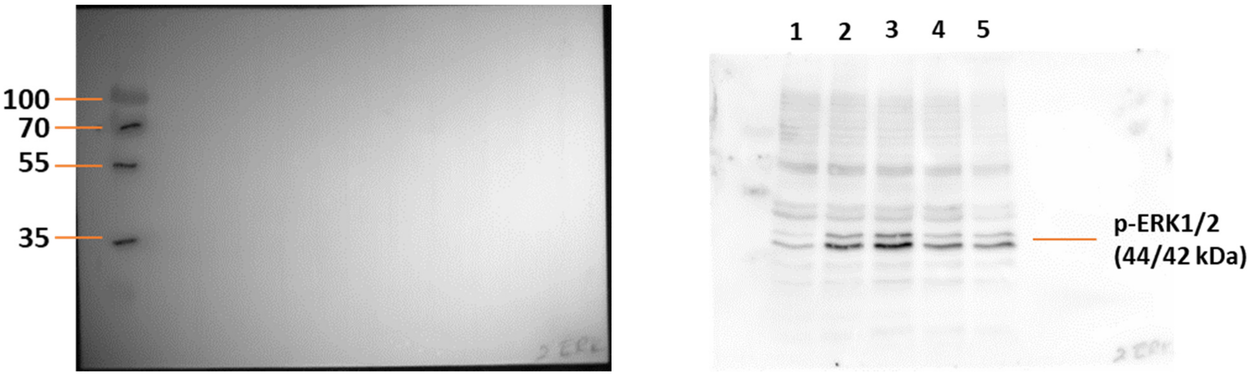

ERK

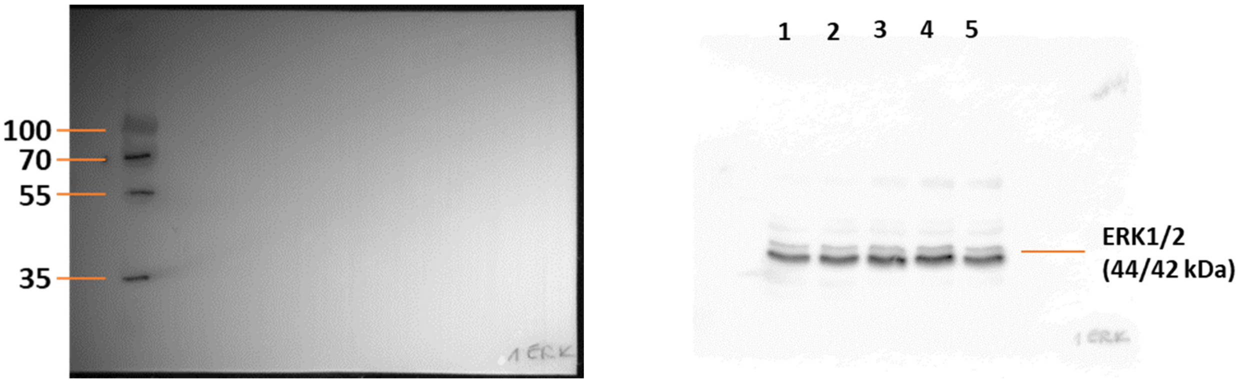

β-actin

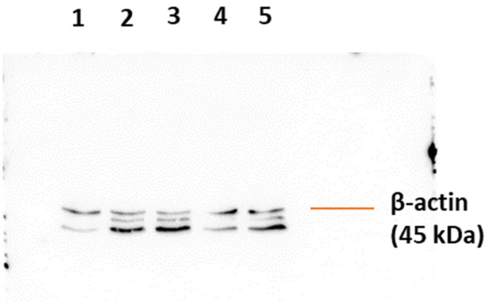

1 **untreated control**
2 **10 µM Ach**
3 **0.1 µM darifenacin + 10 µM Ach**
4 **1 µM darifenacin + 10 µM Ach**
5 **10 µM darifenacin + 10 µM Ach**

2018-04-29

p-ERK

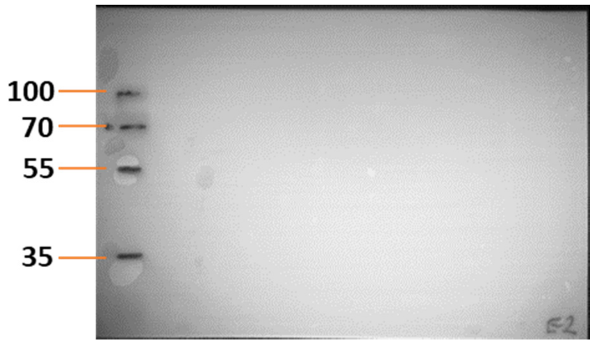

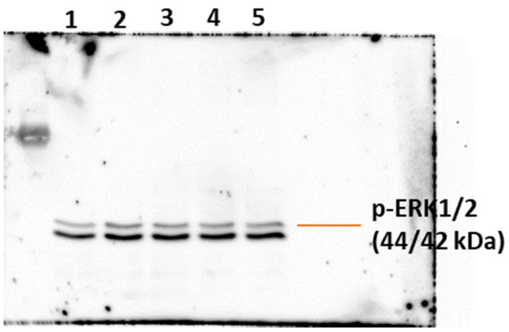

ERK

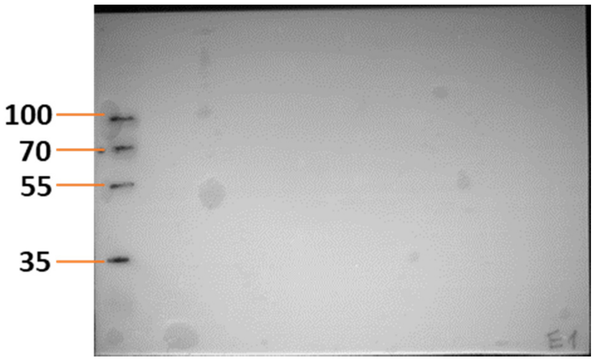

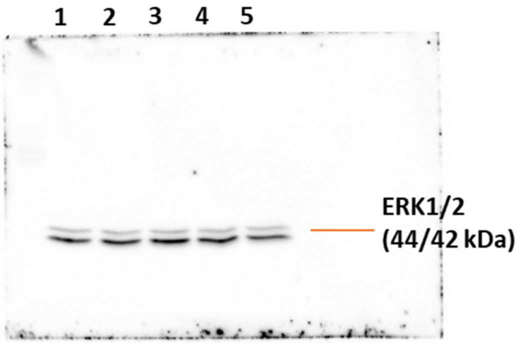

β-actin

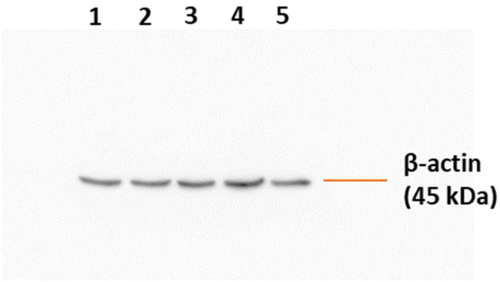

1 untreated control
2 10 µM Ach
3 0.1 µM darifenacin + 10 µM Ach
4 1 µM darifenacin + 10 µM Ach
5 10 µM darifenacin + 10 µM Ach

2018-06-08

p-ERK

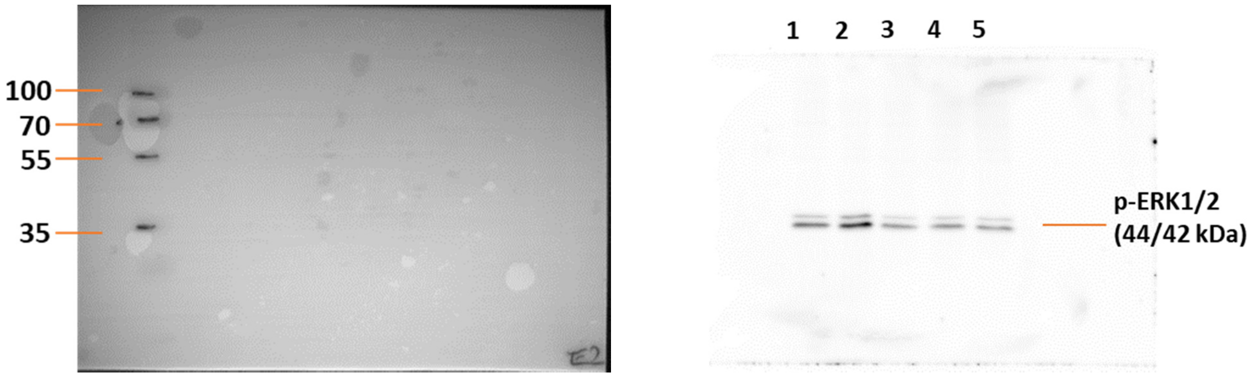

ERK

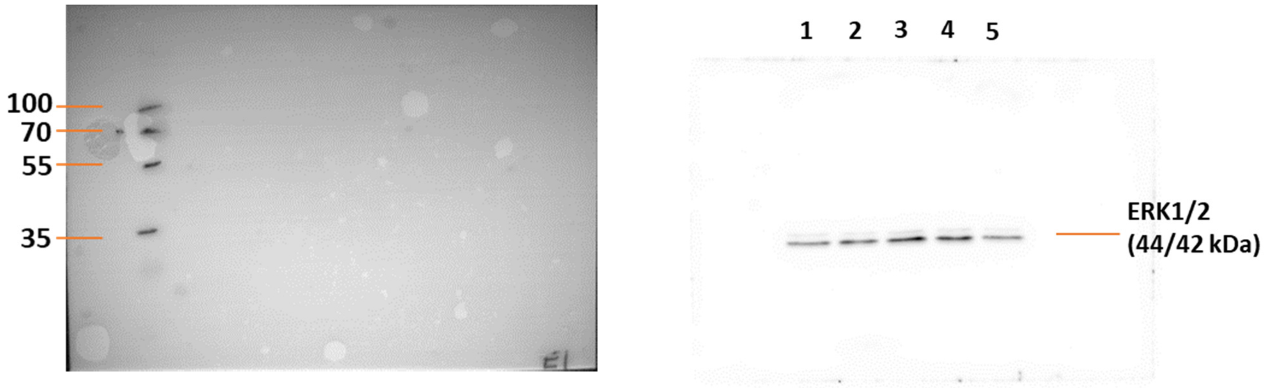

β-actin

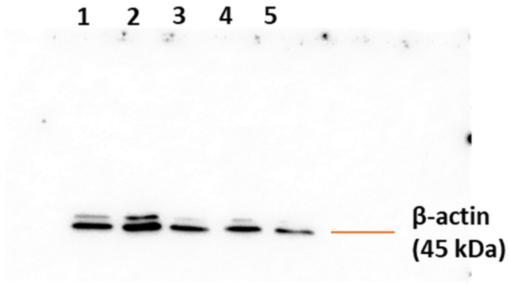

1 untreated control
2 10 µM Ach
3 0.1 µM darifenacin + 10 µM Ach
4 1 µM darifenacin + 10 µM Ach
5 10 µM darifenacin + 10 µM Ach

2017-02-05

p-Sarc

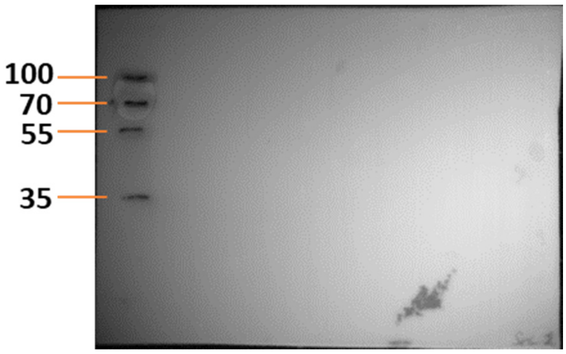

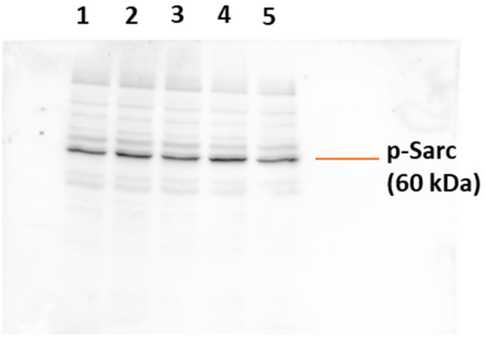

Sarc

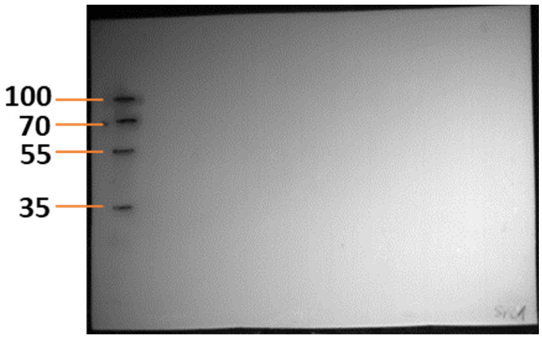

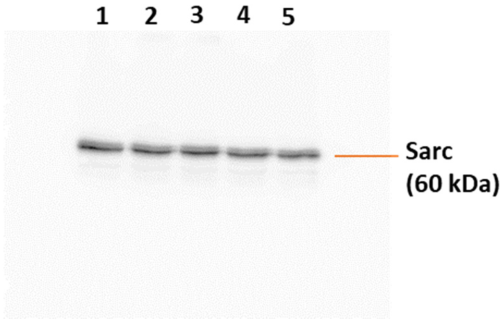

β-actin

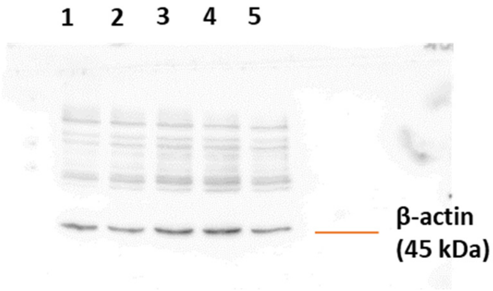

1	untreated control
2	10 μM Ach
3	0.1 μM darifenacin + 10 μM Ach
4	1 μM darifenacin + 10 μM Ach
5	10 μM darifenacin + 10 μM Ach

2017-02-19

p-Sarc

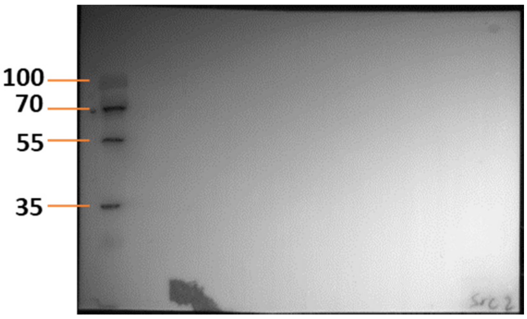

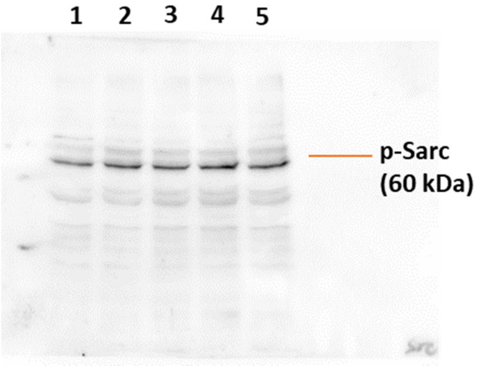

Sarc

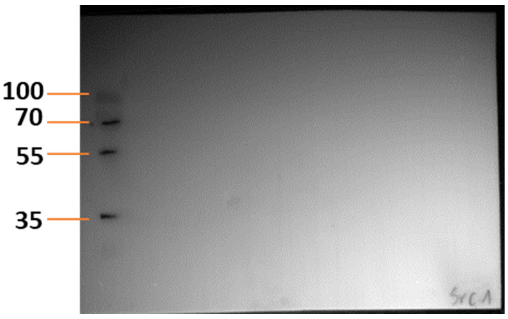

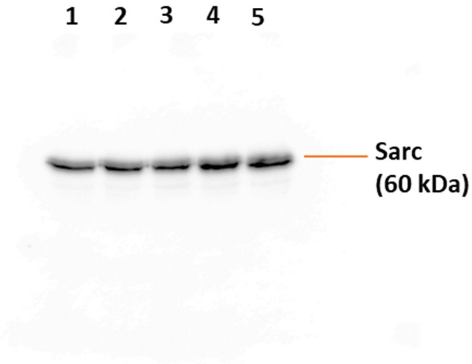

β-actin WB

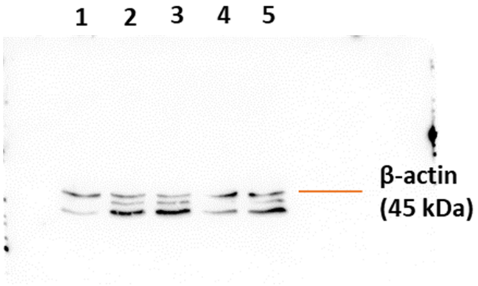

1	untreated control
2	10 µM Ach
3	0.1 µM darifenacin + 10 µM Ach
4	1 µM darifenacin + 10 µM Ach
5	10 µM darifenacin + 10 µM Ach

2017-04-29

p-Sarc

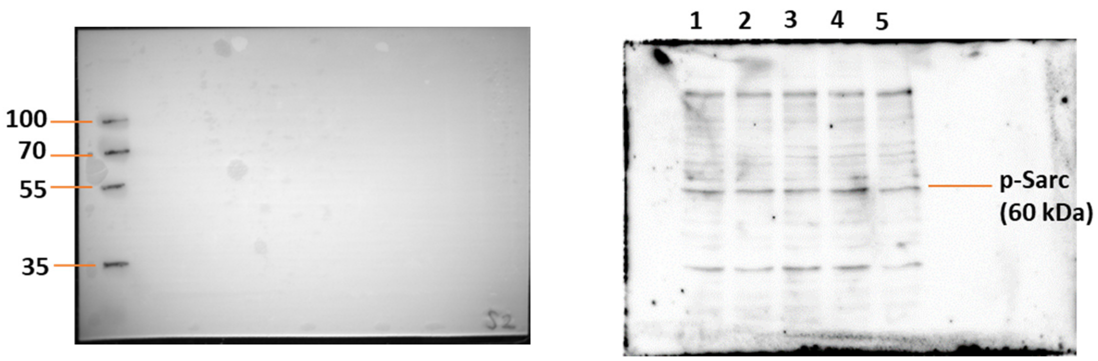

Sarc

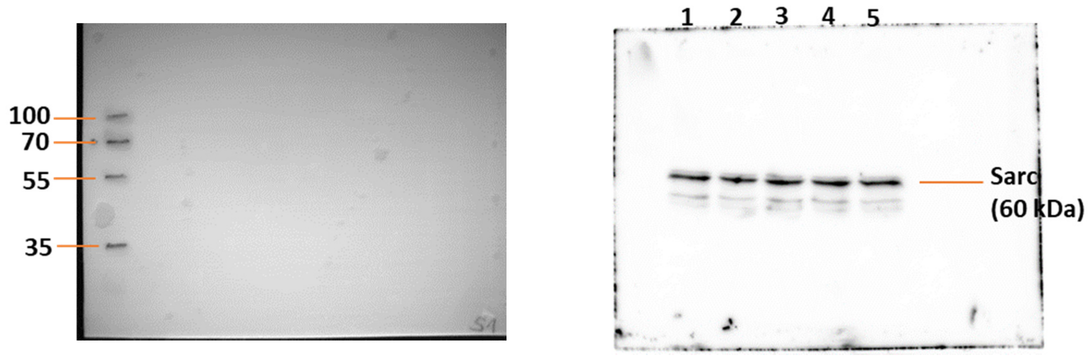

β-actin

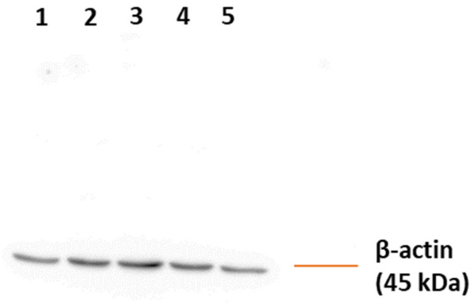

1 untreated control
2 10 µM Ach
3 0.1 µM darifenacin + 10 µM Ach
4 1 µM darifenacin + 10 µM Ach
5 10 µM darifenacin + 10 µM Ach

2018-01-21

Act

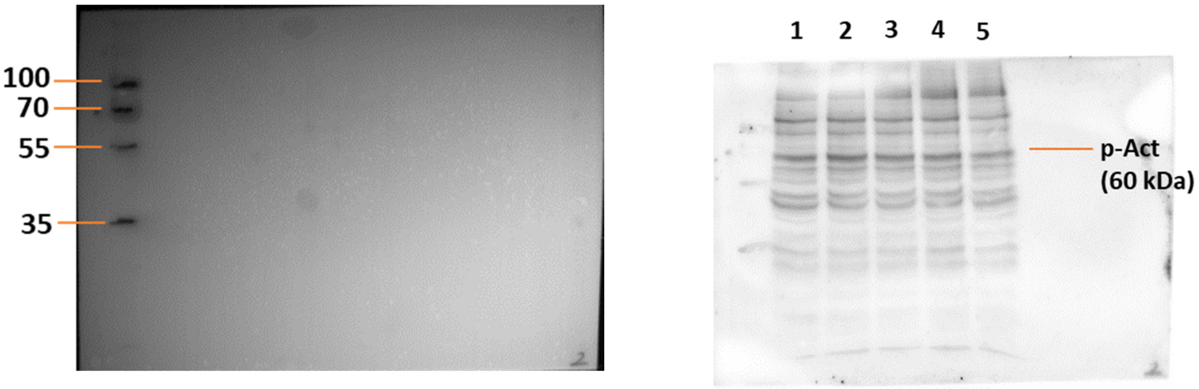

p-Act

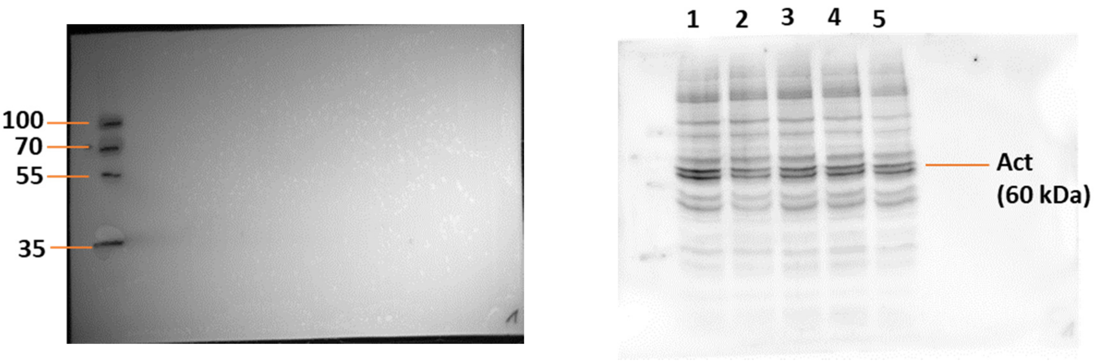

β-actin

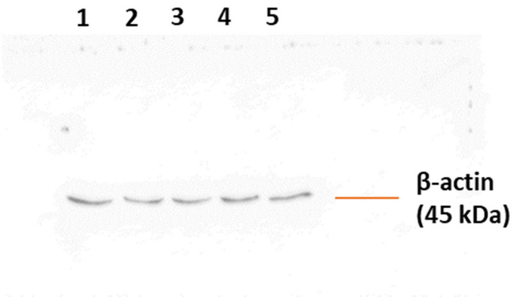

1 untreated control
2 10 µM Ach
3 0.1 µM darifenacin + 10 µM Ach
4 1 µM darifenacin + 10 µM Ach
5 10 µM darifenacin + 10 µM Ach

2018-06-11

p-Act

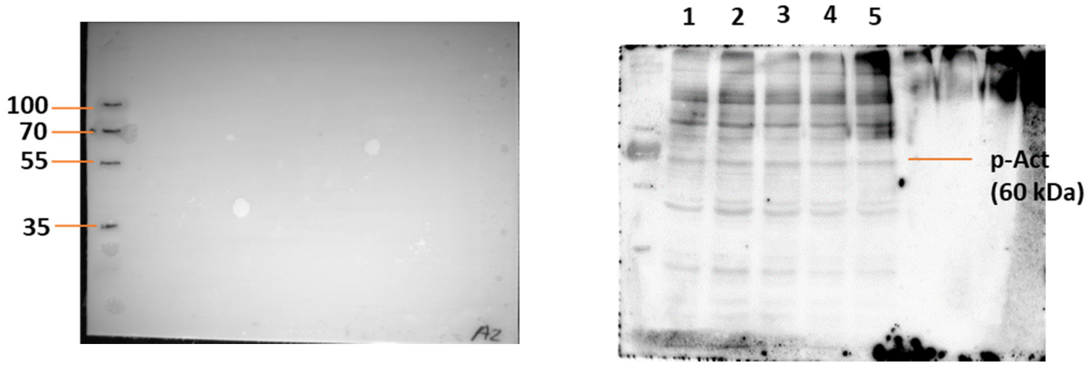

Act

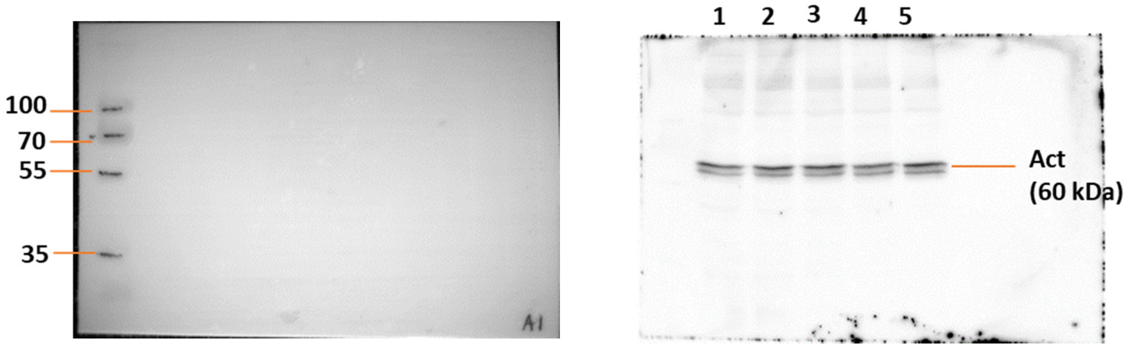

β-actin (= ERK 2018-06-08)

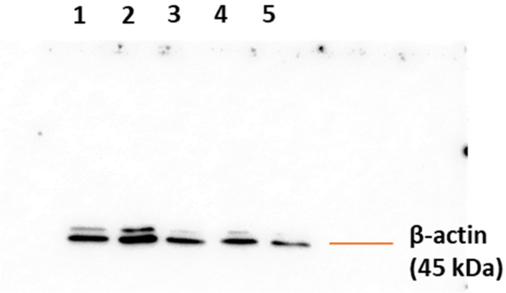

1 **untreated control**
2 **10 µM Ach**
3 **0.1 µM darifenacin + 10 µM Ach**
4 **1 µM darifenacin + 10 µM Ach**
5 **10 µM darifenacin + 10 µM Ach**

2018-06-17

p-Act

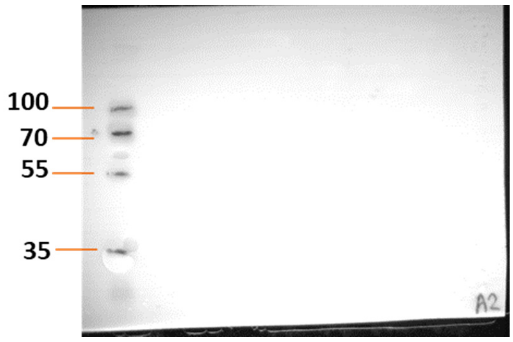

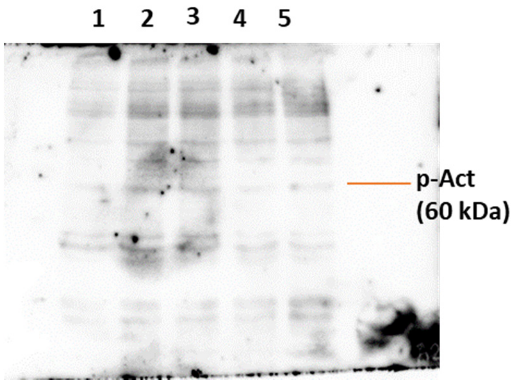

Act

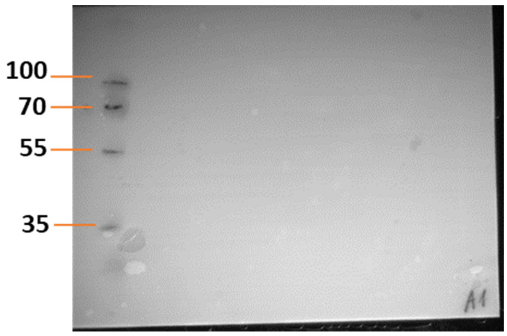

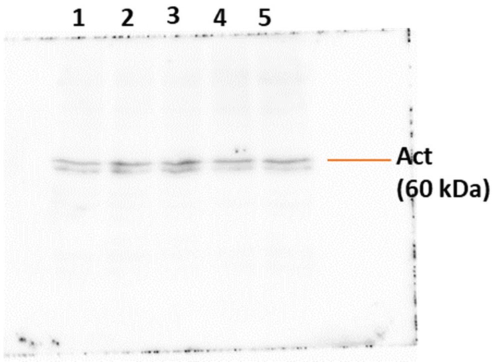

β-actin

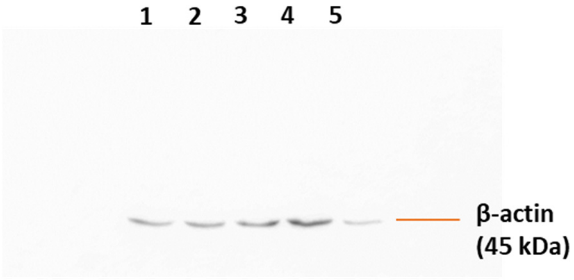

1 untreated control
2 10 µM Ach
3 0.1 µM darifenacin + 10 µM Ach
4 1 µM darifenacin + 10 µM Ach
5 10 µM darifenacin + 10 µM Ach

2018-06-20

p-Act

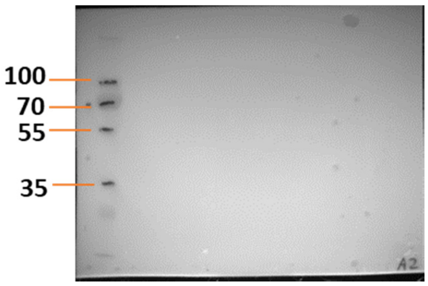

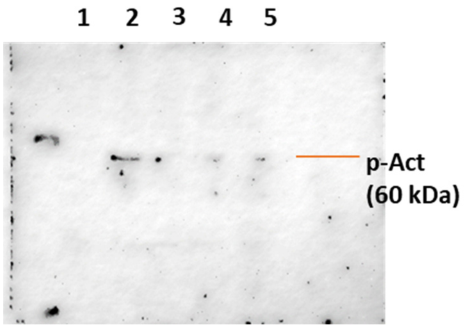

Act

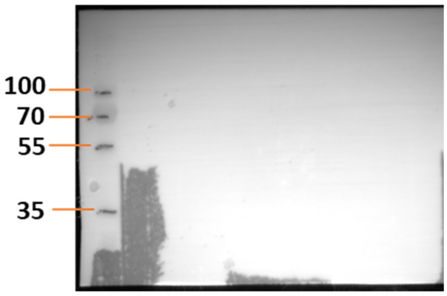

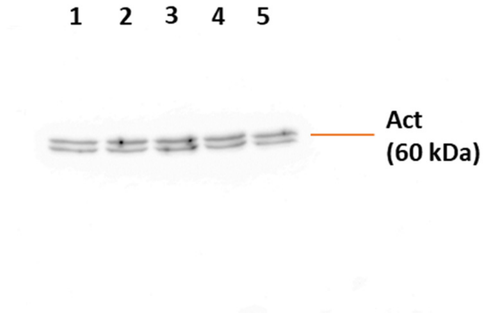

β-actin

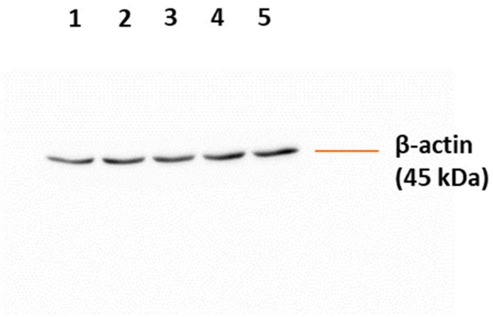

1 untreated control
2 10 µM Ach
3 0.1 µM darifenacin + 10 µM Ach
4 1 µM darifenacin + 10 µM Ach
5 10 µM darifenacin + 10 µM Ach

2018-04-29

p-p38

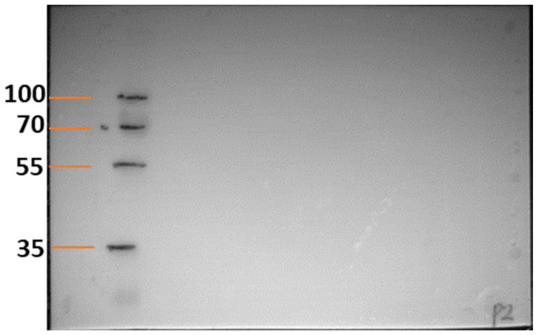

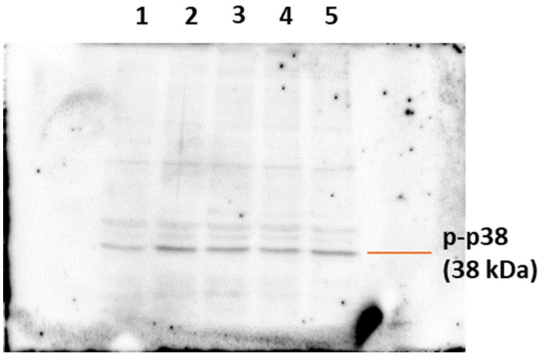

p38

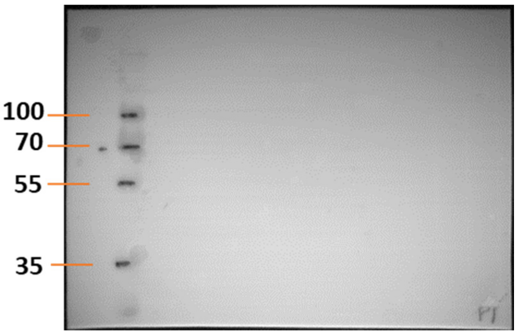

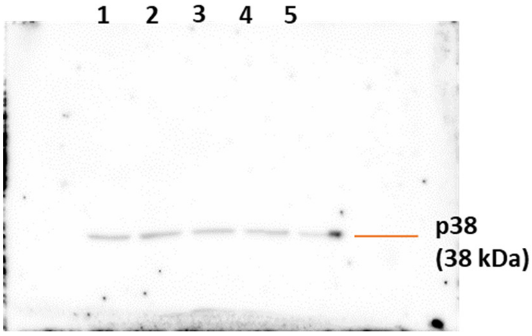

β-actin (= 2018-04-29 Sarc)

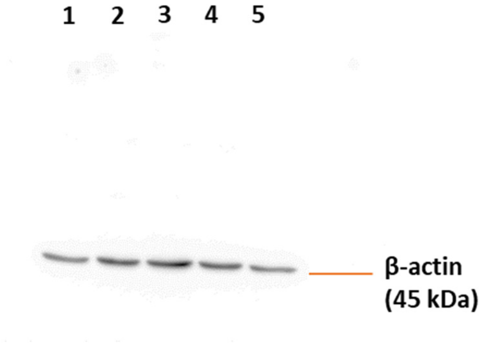

1 untreated control
2 10 µM Ach
3 0.1 µM darifenacin + 10 µM Ach
4 1 µM darifenacin + 10 µM Ach
5 10 µM darifenacin + 10 µM Ach

2018-05-14

p-p38

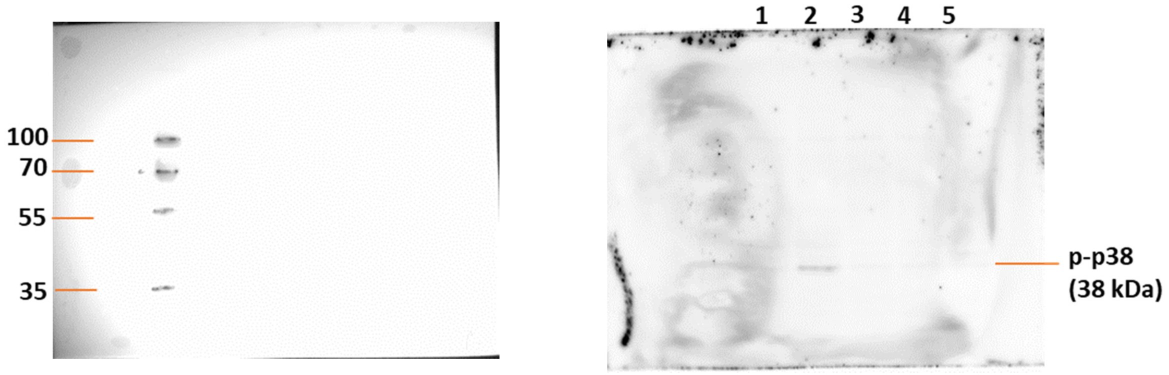

p38

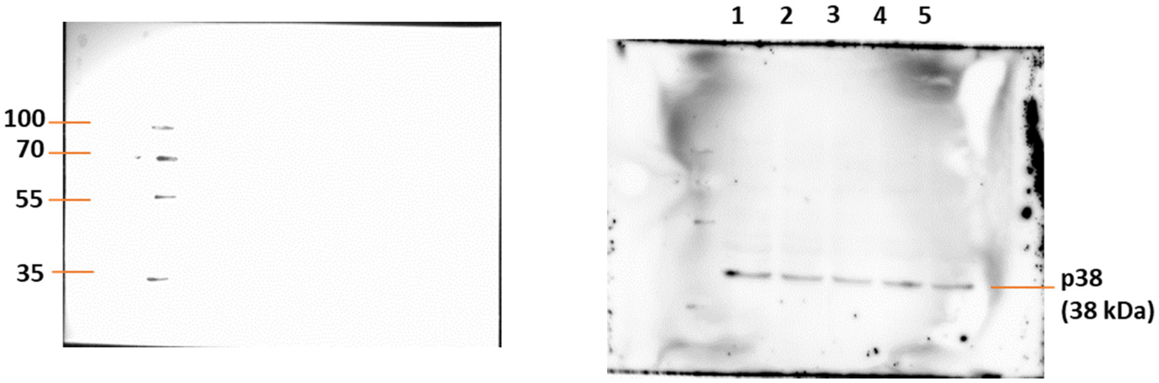

β-actin

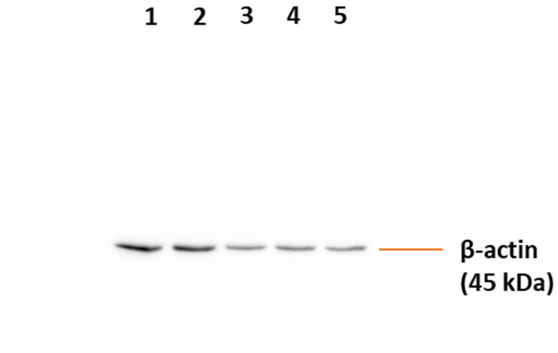

1	untreated control
2	10 µM Ach
3	0.1 µM darifenacin + 10 µM Ach
4	1 µM darifenacin + 10 µM Ach
5	10 µM darifenacin + 10 µM Ach

2018-06-17

p-p38

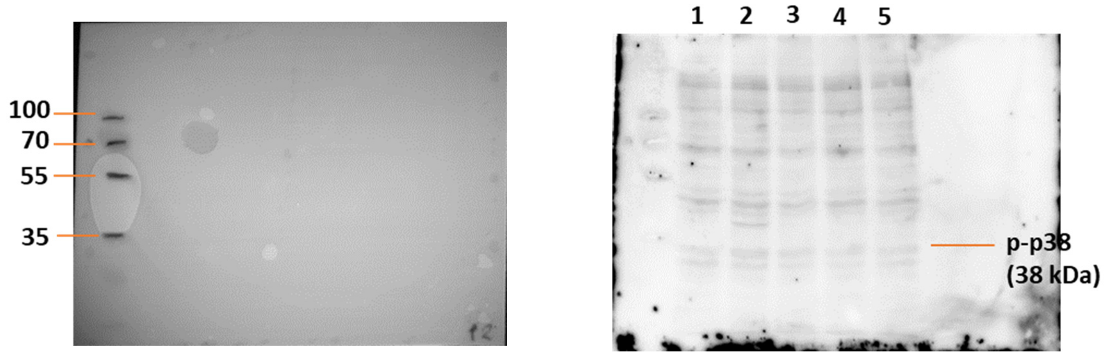

p38

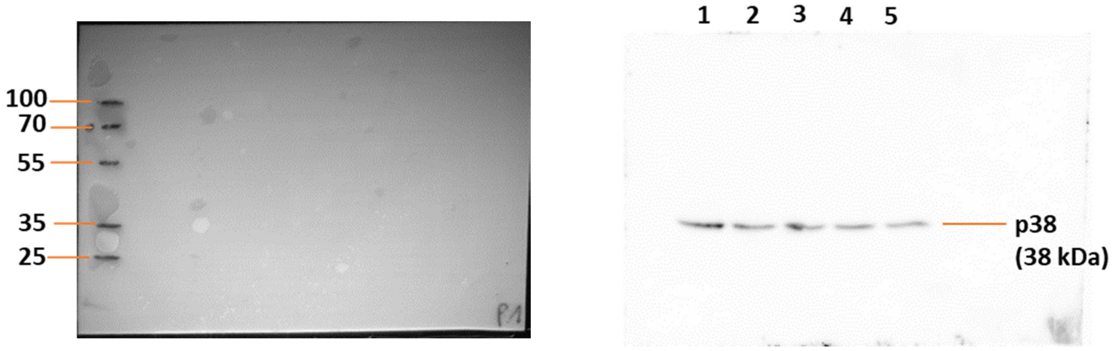

β-actin

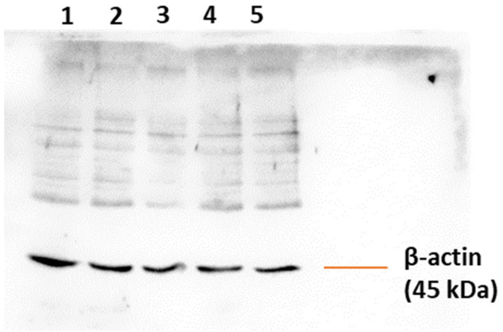

1 untreated control
2 10 µM Ach
3 0.1 µM darifenacin + 10 µM Ach
4 1 µM darifenacin + 10 µM Ach
5 10 µM darifenacin + 10 µM Ach

2018-07-09

p38

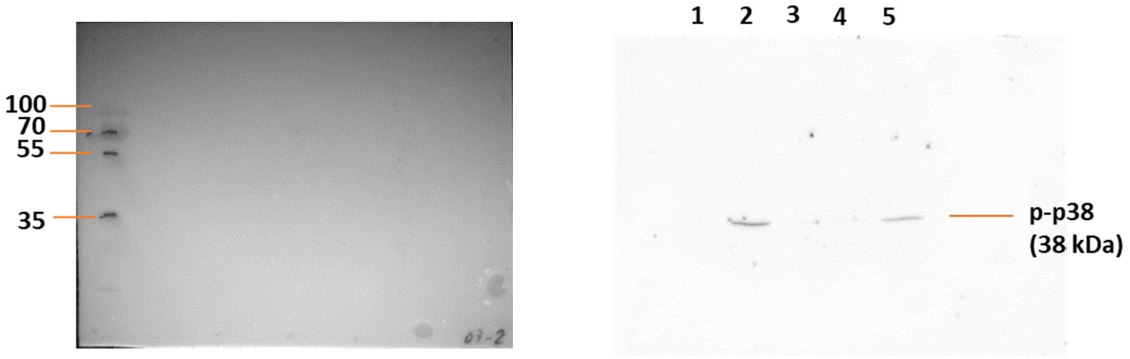

p-p38

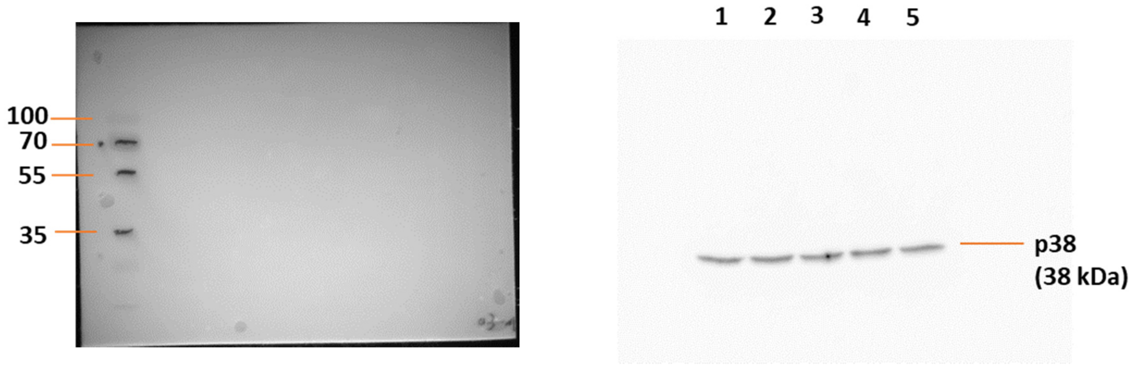

β-actin

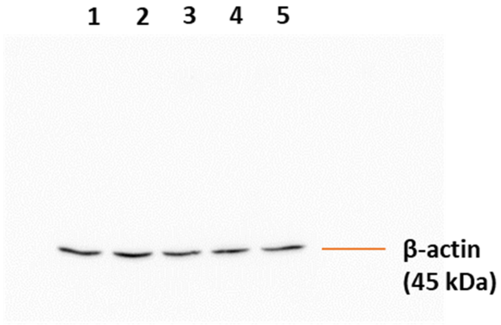

1 untreated control
2 10 µM Ach
3 0.1 µM darifenacin + 10 µM Ach
4 1 µM darifenacin + 10 µM Ach
5 10 µM darifenacin + 10 µM Ach

Figure S1. Blots relative to Figure 2a**.**
